# Using ChatGPT to Predict Cancer Predisposition Genes: A Promising Tool for Pediatric Oncologists

**DOI:** 10.7759/cureus.47594

**Published:** 2023-10-24

**Authors:** Iyad Sultan, Haneen Al-Abdallat, Zaina Alnajjar, Layan Ismail, Razan Abukhashabeh, Layla Bitar, Mayada Abu Shanap

**Affiliations:** 1 Department of Pediatrics, King Hussein Cancer Center, Amman, JOR; 2 Department of Medicine, University of Jordan, Amman, JOR; 3 Department of Medicine, Hashemite University, Zarqa, JOR; 4 Department of Cell Therapy and Applied Genomics, King Hussein Cancer Center, Amman, JOR; 5 Department of Pediatric Oncology, King Hussein Cancer Center, Amman, JOR

**Keywords:** chatgpt, genes, cancer, oncology, pediatrics

## Abstract

Background: Determining genetic susceptibility for cancer predisposition syndromes (CPS) through cancer predisposition genes (CPGs) testing is critical in facilitating appropriate prevention and surveillance strategies. This study investigates the use of ChatGPT, a large language model, in predicting CPGs using clinical notes.

Methods: Our study involved 53 patients with pathogenic CPG mutations. Two kinds of clinical notes were used: the first visit note, containing a thorough history and physical exam, and the genetic clinic note, summarizing the patient's diagnosis and family history. We asked ChatGPT to recommend CPS genes based on these notes and compared these predictions with previously identified mutations.

Results: Rb1 was the most frequently mutated gene in our cohort (34%), followed by *NF1* (9.4%), *TP53* (5.7%), and *VHL* (5.7%). Out of 53 patients, 30 had genetic clinic notes of a median length of 54 words. ChatGPT correctly predicted the gene in 93% of these cases. However, it failed to predict *EPCAM* and *VHL* genes in specific patients. For the first visit notes (median length: 461 words), ChatGPT correctly predicted the gene in 64% of these cases.

Conclusion: ChatGPT shows promise in predicting CPGs from clinical notes, particularly genetic clinic notes. This approach may be useful in enhancing CPG testing, especially in areas lacking genetic testing resources. With further training, there is a possibility for ChatGPT to improve its predictive potential and expand its clinical applicability. However, additional research is needed to explore the full potential and applicability of ChatGPT.

## Introduction

Cancer in children is generally considered a rare and sporadic event. However, recent reports using genome-scale germline sequencing of pediatric cancer cohorts indicate that at least 10% of these individuals carry a hereditary mutation in well-known genes associated with increased cancer susceptibility. This percentage is probably underestimated because there are also patients who fulfill clinical criteria for a cancer predisposition syndrome (CPS) but lack identifiable germline mutation in the known gene(s) currently associated with those conditions [1-3].

It is widely accepted that various factors such as pathogenic variants in cancer predisposition genes (CPGs), epigenetic factors, and abnormalities in other gene products are widely acknowledged to play a role in the development of childhood cancers [4,5]. Studies conducted in the field of pediatrics provide evidence indicating that enhanced monitoring and timely identification of cancer in individuals harboring a hereditary mutation associated with cancer predisposition can potentially lead to better outcomes [6-8]. Identifying such patients has the potential to facilitate adjustments in therapy when there is an increased risk of treatment-related toxicity or resistance in the presence of a specific syndrome. Furthermore, the implementation of targeted screening programs tailored to specific syndromes can contribute to the early detection of additional independent malignancies. In addition to the affected individuals, it may be advisable to consider cancer surveillance for their relatives, and the detection of a genetic mutation can offer opportunities for reproductive counseling [9,10].

The utilization of next-generation sequencing has revolutionized the application of tumor and germline genomic testing in the treatment of pediatric cancer patients. Although hereditary cancer predisposition syndromes are rare, there is a possibility that pediatric oncologists may come across them during their medical practice. However, many providers in the field of pediatric oncology lack the confidence to identify specific cancer predisposition genes and effectively interpret and utilize genomic results from tumor or germline testing in the care of their patients [11].

Recently, artificial intelligence (AI) programs and large language models (LLMs) like ChatGPT have emerged as promising tools with many potential uses in medical practice [12-14,15]. ChatGPT has shown the potential to streamline clinical workflows, potentially leading to cost savings and heightened efficiency in healthcare delivery. Its transformative potential in healthcare is vast, enhancing diagnostics, predicting disease risk and outcomes, and even assisting in drug discovery [16].

ChatGPT has made notable advancements in clinical and translational medicine, notably within the field of oncology. Research has demonstrated its potential for interpreting next-generation sequencing reports, analyzing patient records, and suggesting suitable clinical trials for cancer patients [17,18]. ChatGPT has even showcased the ability to provide quality and empathetic responses to patient inquiries in online forums, surpassing physician responses in terms of quality and empathy [19].

The path to integrating AI into pediatric medicine and cancer predisposition detection is not without its challenges [20]. The need for stringent regulatory standards and a balance between innovation and human judgment is critical. This study aims to further the existing research on the application of AI in cancer diagnosis and treatment and explores the potential of ChatGPT as a tool to predict CPGs based on clinical notes.

## Materials and methods

All patients included in this study had undergone genetic testing for targeted 84 cancer genes, and genomic DNA obtained from the peripheral blood was sent out to an Invitae Lab in the United States; submitted samples were enriched for targeted regions using a hybridization-based protocol and sequenced using Illumina technology, and all targeted regions are sequenced with ≥50x depth or are supplemented with additional analysis.​ The study cohort was selected by identifying 53 patients who carried pathogenic mutations. Patients were tested for CPGs if they fulfilled at least one of McGill Interactive Pediatric OncoGenetic Guidelines (MIPOGG) or Jongmans' criteria; these are tools that recognize patients with potential cancer predisposition syndrome based on (a) a clinical phenotype, where ordering was based on physical examination findings; (b) family history of cancer, where more than one first- or second-degree relative had cancer at an age younger than 50 years; and (c) specific tumor subtype, where ordering was based on a tumor subtype association [2,3].

For the purpose of our analysis, we used two types of clinical notes: the notes from the first visit, which encapsulated a comprehensive history and physical examination, and the genetic clinic notes, which provided a summary of the patient's diagnosis and family history. It is worth noting that we meticulously screened these notes to ensure they did not contain direct indicators of the diagnosis, such as a known TP53 mutation in the family.

Our next step involved using the AI model, ChatGPT, by posing the question, "What genes do you recommend testing for this patient?" Each clinical note was then fed into the system separately. The suggestions produced by ChatGPT were then compared with previously identified mutations. A prediction was deemed correct if the diagnosed gene was present within the list of genes suggested by ChatGPT. Notably, this process was facilitated using the free version of ChatGPT3.5, available at https://www.openai.com/ (Version GPT-3.5) (Software) on March 18, 2023. Finally, we ensured that the study complied with ethical guidelines and received approval from the King Hussein Cancer Center's Institutional Review Board (IRB).

## Results

Demographics, diagnosis, and genetic mutations

We analyzed 53 pediatric patients who underwent genetic testing. Among these, 60% were male (n=32) and 40% were female (n=21). The median age at the time of cancer diagnosis was three years, as shown in Table 1.

**Table 1:** Characteristics of patients *Others include gastrointestinal tumors, gynecological tumors, kidneys tumors, and muscle tumors. IQR: interquartile range.

Characteristics	N (%)
Gender	
Male	32 (60%)
Female	18 (40%)
Age	
Median (IQR)	3 (1.5-10)
Inheritance	
Heterozygous	36 (68%)
Homozygous	11 (21%)
Possibly mosaic	6 (11%)
Cancers	
Hematologic malignancy	5 (9%)
Retinoblastoma	18 (34%)
Brain tumors	14 (26%)
*Others	16 (30%)

**Figure 1:**
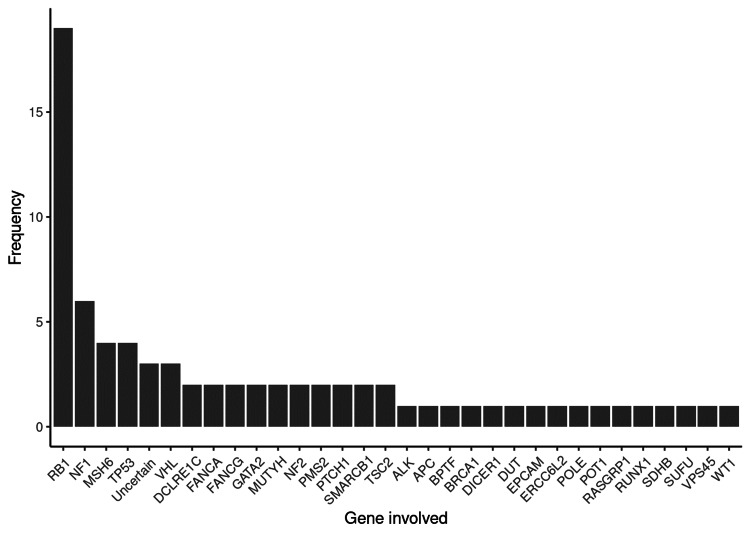
Genes involved and their frequency

Retinoblastoma was the most frequently diagnosed cancer, with unilateral and bilateral presentations observed in 17% (n=9) and 15% (n=8) of patients, respectively. Additional cancer types included acute myeloid leukemia (AML), atypical teratoid/rhabdoid tumor (ATRT), high-grade glioma, and medulloblastoma. A substantial majority of patients (89%) were diagnosed with cancer predisposition syndromes (CPS). The *RB1* gene mutation was the most prevalent, found in 34% (n=18) of patients. This was followed by mutations in the *NF1* gene in five patients (9.4%), *TP53* in three patients (5.7%), and VHL in three patients (5.7%). These findings are summarized in Figure 1.

With regards to inheritance, 68% of patients displayed heterozygous inheritance, while 21% were homozygous and the remaining 11% were possibly mosaic. All patients fulfilled the McGill Pediatric OncoGenetic Guidelines (MIPPOG), with 70% meeting criteria 3, 53% meeting criteria 2, and 45% meeting criteria 1. As per Figure 1, it highlights the involved genes and their frequencies.

Genetic note analysis

We examined 30 available genetic clinic notes, where ChatGPT correctly predicted the pathogenic gene in 93% (28 out of 30) of cases. The AI model failed to predict two genes, *EPCAM* (in a patient with colorectal cancer) and *VHL* (in a patient with acute lymphocytic leukemia (ALL) and Ewing sarcoma). The word count for each genetic note ranged from five to 1,022, with a median of 54 words (interquartile range, 38-109). On average, each genetic note suggested 3.4 genes, ranging from one to 12 genes.

First visit clinic note analysis

Out of 47 notes, ChatGPT correctly predicted the pathogenic gene in 64% (30 out of 47) of cases. The median word count for the initial clinic visit note was 460.5 words (interquartile range, 272-611). On average, each first visit note suggested 4.5 genes, with a range from one to 11 genes.

## Discussion

ChatGPT has generated interest in healthcare research owing to its potential benefits in academia, disease diagnosis, prognosis, and patient care [16]. Its role in predicting genetic mutations across different cancers may contribute to better patient outcomes through efficient gene detection tests and expedited diagnoses [21,22]. This study explores ChatGPT's capacity to predict CPGs in pediatric cancer patients using genetic and first-visit clinic notes from 53 patients who underwent genetic testing at our center.

Our results indicate that ChatGPT, when equipped with genetic clinic notes, shows the promising predictive output. It achieved a correct result rate of 93% (28 out of 30) when using genetic clinic notes and 64% (30/47) when using first-visit clinic notes. The discrepancy in the result can be attributed to the nature of the first-visit notes. While these notes provide a summary of the patient's clinical presentation of cancer and a detailed family history, they often lack the final diagnosis of cancer, as it requires the biopsy of the tumor and histopathology analysis. Additionally, certain features that predict cancer predisposition and may evolve later, such as chemo-/radiosensitivity and the possibility of second cancer, are also not consistently captured in the first-visit notes. On the other hand, physicians' insight about potential CPS is summarized in genetic clinic notes and enhances AI models' predictive abilities. Notably, ChatGPT failed to predict *EPCAM* and *VHL* genes in genetic clinic notes. The presence of a *VHL* gene mutation in a patient with ALL and Ewing sarcoma is unusual and might indicate genetic instability or the driver mutation is not in included our targeted 84 genes panel. On the other hand, *EPCAM*, a gene associated with biallelic mismatch disorder, should have been predicted. Interestingly, when queried directly about this syndrome's associated genes, ChatGPT correctly listed *EPCAM*. However, the intrinsic opaqueness of these learning models creates complexity in comprehending their decision-making process, making it challenging for humans to clearly understand, analyze, or fully comprehend.

In Pediatric Cancer Genome Project (PCGP), germline mutations in CPGs were identified in 8.5% of the children and adolescents with cancer; the most commonly mutated genes in the affected patients were *TP53*, followed by *APC*, *BRCA2*, *NF1*, *PMS2*, *RB1*, and *RUNX1* [4]. In contrast, the most frequently mutated genes in our patients were *RB1*, followed by *NF1*, *TP53*, and *VHL*. The majority of detected genes were classified as pathogenic with a heterozygous autosomal dominant inheritance mode. This highlights the need for experts in this field to facilitate evidence-based practices and to expand counseling resources [23]. This need becomes even more pronounced in low- and middle-income countries (LMICs), where the cost of genetic services serves as a significant barrier [24]. Given the complexity of interpreting genetic data and its impact on clinical decision-making, the involvement of medical experts in guiding the ChatGPT process becomes paramount, emphasizing their importance in ensuring accurate and effective outcomes.

Our study has several limitations. The notes used were not standardized, and the structure of our first-visit notes may have affected prediction quality. We utilized ChatGPT3.5 without comparison to other models such as ChatGPT-4, which has now access to live internet browsing, that could improve ChatGPT genetic predictions in the future and which suggests an integration model of ChatGPT and medical science to predict personalized patient's care [25]. Despite these limitations, our findings endorse the application of ChatGPT in investigating the genetic underpinnings of childhood cancer, a complex and expertise-demanding area. This is especially critical in LMICs, where access to genetic experts and counselors is limited [24]. It is also important to acknowledge that ChatGPT, like other AI models, has its own set of limitations and concerns regarding ethical considerations and potential bias. It is essential to recognize these limitations and exercise caution when interpreting and relying solely on ChatGPT's responses in a medical context [20].

## Conclusions

In conclusion, our study demonstrates compelling evidence and promising potential of ChatGPT in predicting CPGs from clinical notes, particularly in the field of genetic clinics. This innovative approach lays the foundation for future investigations in enhancing CPG testing and implementation process, especially in areas lacking genetic testing resources. Although our study represents an initial exploration, it has shed light on the predictive potentials of ChatGPT; nevertheless, further research and training can refine and improve the predictive accuracy and clinical applicability of ChatGPT. Overall, ChatGPT holds promise as a tool for improving healthcare decision-making by correctly predicting CPGs from clinical notes, and with continued advancements, it has the potential to significantly enhance healthcare outcomes and patient care.
